# Supplementary material for: Developmental time windows for axon growth influence neuronal network topology
Source: Biol Cybern. 2015 Jan 30;109(2):275–86. doi: 10.1007/s00422-014-0641-3 (PMC4366563; doi:10.1007/s00422-014-0641-3)
Supplement: Supplementary file 1 — Supplementary material 1 (pdf 13824 KB) [file 422_2014_641_MOESM1_ESM.pdf]

Supplementary Material

A1. Discrete and Continuous search mode

Discrete and continuous search mode were compared, however, as there was no qualitative difference between the results using discrete and continuous search mode (for quantitative differences from a modelling perspective see A2.2), we show results with continuous search mode without losing generality in the main text. For discrete search mode, the growth cone of a neuron proceeded along its given direction by one unit Euclidean distance per each iteration time. For continuous search mode, neurons were assumed to have the same average speed as for discrete search mode (or one unit distance per unit time) but were allowed to establish synapses at real number distance, hence continuous search mode. Connectible neurons were found along a virtual direction of axon growth by computing the distance between neurons and the axonal growth line. In other words, check the whole axonal direction at once, to see if it ever comes close to dendrites, or encountering a neuron sphere.

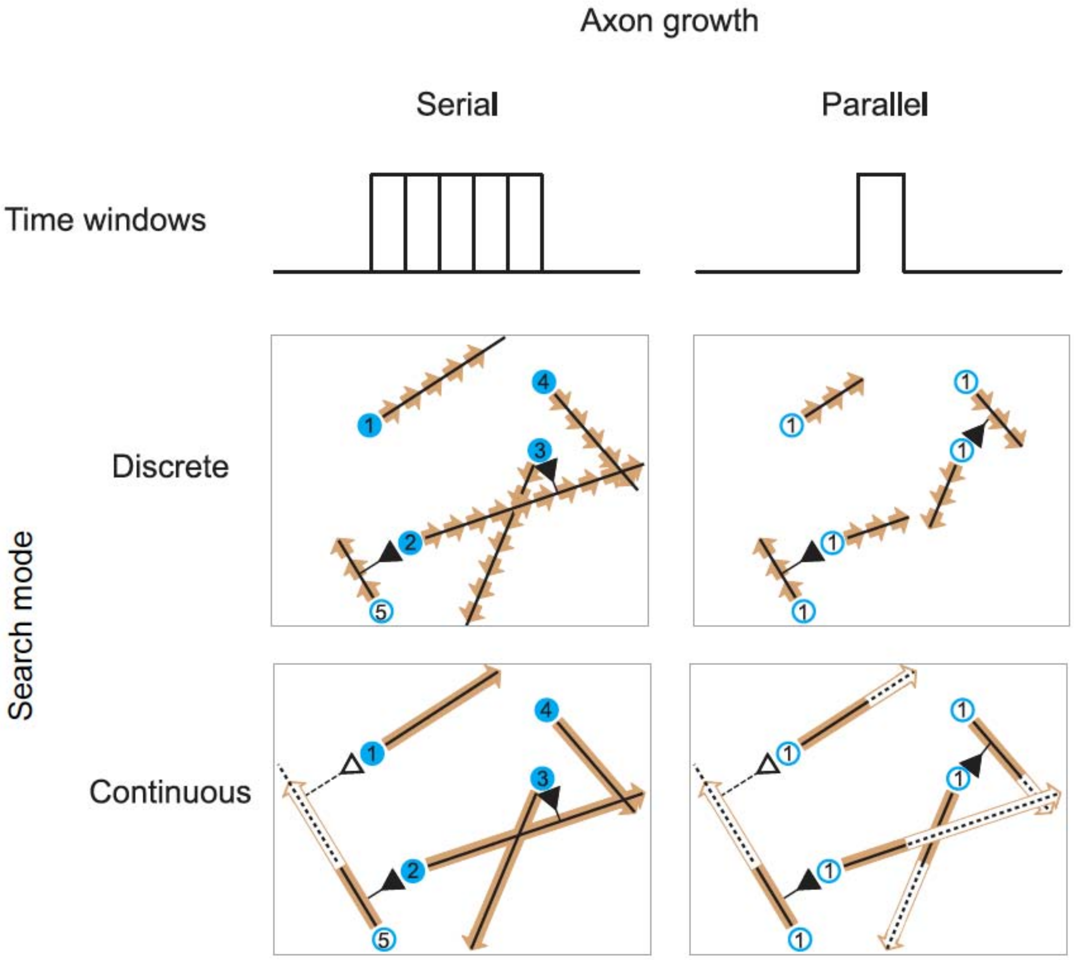

Figure A1. Simulation setting: serial vs. parallel growth and discrete vs. continuous search mode. Serial growth: neurons take turns to grow axons. Parallel growth: all neurons start to grow axons simultaneously. Discrete search mode: neurons search for connectible neurons residing within certain proximity at every discrete time step. Continuous search mode: neurons examine all possible target neurons to establish synapses using simplified ray tracing method. Blue circles: solid circles denote neurons that finished axon growth and empty circles represent neurons that are active. Numbers in the circles represent the sequence of growth. Black triangles: synapses, Black solid line: axons, Black dashed lines: future axon growth path, Short arrows: symbols for discrete growth, Long arrows: continuous growth.

A2. Morphological properties

A2.1 Potential synapses and established synapses

Neuron might miss a connectible neuron in discrete search mode because it establishes synapses only at discrete time steps along the direction (neglected space, Figure A2). At the same time, it is also possible to find the same spot multiple times because the same neuron is likely to be within proximity again after one unit step away (multiple detection space Figure A2). As we did not allow neurons to make multiple synapses with the same neuron in the simulated networks, the total number of potential synaptic places was always larger in continuous search mode than in discrete search mode (Table A2a), which also applied to the number of established synapses (Table A2b). In contrast to the topological and spatial properties (Section 3.1), there was no difference in the number of potential synapses between serial and parallel growth since the placement of neurons and the growth directions were the same and they were independent of serial and parallel growth scenarios.

Table A2a. Relations between the number of potential synaptic locations. Row (left) ~ Column (right) where ~ is among = , < and >. For example parallel continuous > serial discrete, which reads the number of potential synapses for parallel growth using continuous search mode is larger than that of serial growth using discrete time steps.

	Serial	
Parallel	Continuous	Discrete
Continuous	=	>
Discrete	<	=

Table A2b. Relations between the number of established synapses with limited incoming connections.
Row (left) ~ Column (right) where ~ is among = , < and >.

	Serial	
Parallel	Continuous	Discrete
Continuous	=	>
Discrete	<	≥

A2.2 Discrete vs. continuous search mode and neglected vs. multiple detection

While the previous results addressed the biological role of axon growth time windows, we also looked at the influence of the model used for computer simulations of axon growth. As modelling each growth step (numerical discrete simulation) is computationally expensive, using an analytical approach (continuous simulation) saves computational resources. Using continuous search mode increased the total number of potential synapses that neurons found and also increased the number of established synapses. This is due to the better coverage of the growth cone pathway: for discrete steps, neurons may miss possible synaptic places since they can only search for target neurons at specific time steps. The volume of neglected space for discrete search mode depends on the ratio of unit time step to the length of proximity rule. If the proximity defining 'close enough to form a synapse' is smaller than the size of unit time step, the neglected space expands and if the vicinity covers larger space than the unit time step the neglected space shrinks (Figure A2). On the other hand, if the proximity reaches farther than the half of the unit step, the overlapping space of proximity between previous time step and the next step increased the possibility of detecting the same neuron multiple times (Figure A2). The length of time step is usually determined considering trade-off accuracy of the computation and processing time but here we also need to consider the ratio of time step to proximity criterion, or another trade-off between neglected space and multiple detection space. By adopting continuous search mode, we could reduce processing time, however, it can be only applied to piecewise straight lines when assuming that branching of axons or turning of growth direction does not occur often.

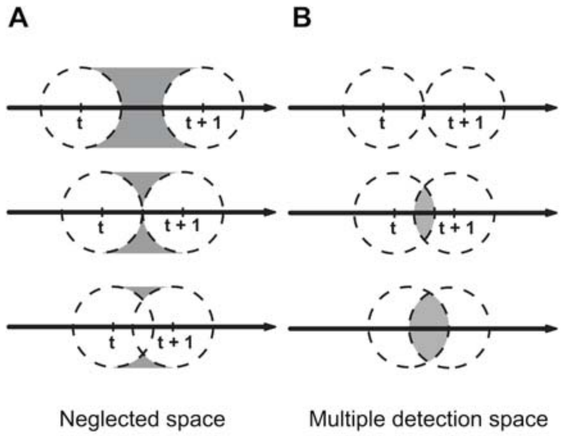

Figure A2. Neglected space and multiple detection when using discrete time steps. (A) Neglected space using discrete time steps growing at times *t* and *t*+1, thick black solid line: growing axon, dashed circles: if a neuron happens to be inside the dashed circle, a synapse can be formed with the neuron. The shaded areas represent neglected space due to discrete time steps. Depending on the ratio between the proximity criterion and the spatial distance covered in one time step, the neglected area can be enlarged and shrunk. (B) Multiple detection of the same neuron using a discrete time step. If a neuron happens to be inside the shaded area, the growth cone can detect the neuron multiple times. Again depending on the ratio between the proximity rule and the magnitude of a time step, the space can be expanded or narrowed.

A3. Bidirectional connectivity

The simulated bidirectional connection probability using inverse sine (e.q.3) was calculated by squaring the connection probability assuming independence among neurons for synapse establishment (Figure A3, black).

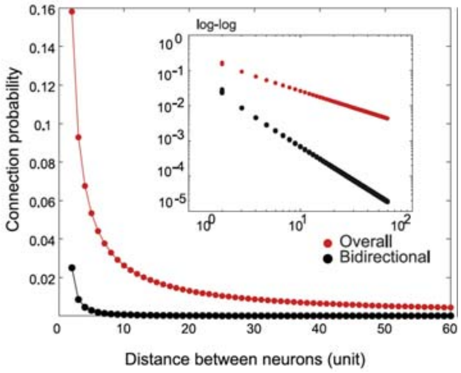

Figure A3. Theoretically generated connection probability and bidirectional connection probability Red: overall connection probability, black: bidirectional connection probability. Inset. Bidirectional connection probability decreased more rapidly with distance between neurons than overall connection probability.

Table A3 Small-worldness

Column: neuron size (radius), row: the number of neurons in the embedding space

Small-worldness is defined by the normalised clustering coefficient divided by the normalised characteristic path length by using rewired networks (See methods). As the rewired networks with neurons having 0.5 radius and 1 maximum incoming connection showed almost zero clustering coefficients, it was not possible to normalise our generated networks with the same condition, therefore we provide 9 conditions out of total 12 conditions in the following tables.

a. Serial growth

	0.603	0.735	0.900
1000	23.3531	15.7397	11.0682
1400	22.2764	17.0195	12.7509
1800	21.3488	17.8679	13.3222

b. Parallel growth

	0.603	0.735	0.900
1000	50.7791	18.8222	11.6412
1400	61.1910	24.5454	13.5337
1800	124.7313	32.4325	15.7965

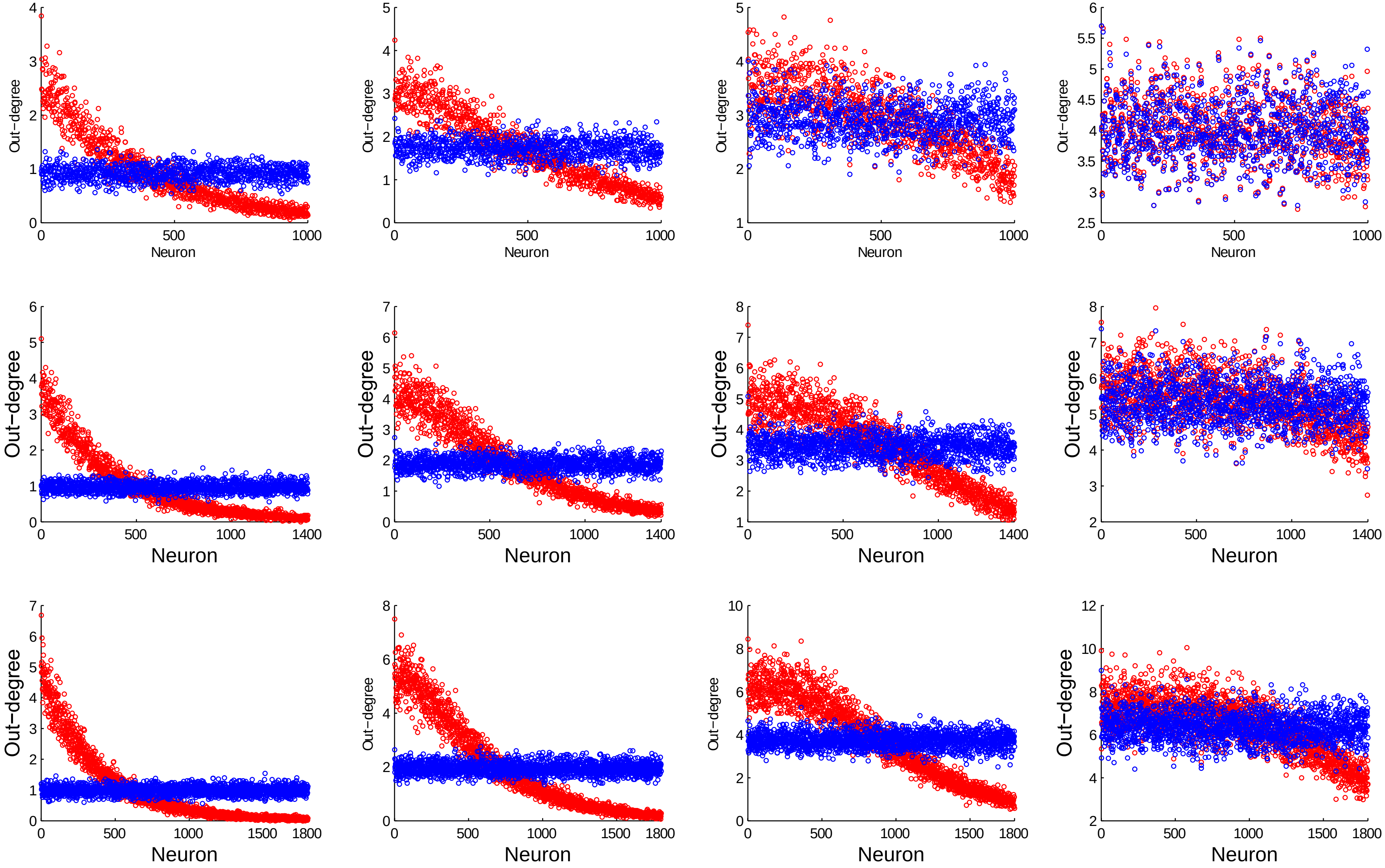

Figure A4. Out-degree Red: serial growth, Blue: parallel growth x-axis: the sequence (order) of neurons starting to grow axons, y-axis: out-degree From the left to the right, the size of neurons increases from 0.5 to 0.9 (0.500, 0.604, 0.735, 0.900) and also the maximum number of incoming connections increases. From top to bottom, the number of neurons increases from 1000 to 1800 by steps of 400. The discrepancy of out-degrees between serial and parallel growth became less obvious for bigger neurons with more incoming connections. For serial growth, neurons started growing axons early on were characterized by higher out-degrees than late starter neurons, whereas the order of growing axons did not influence out-degree distribution for parallel growth.

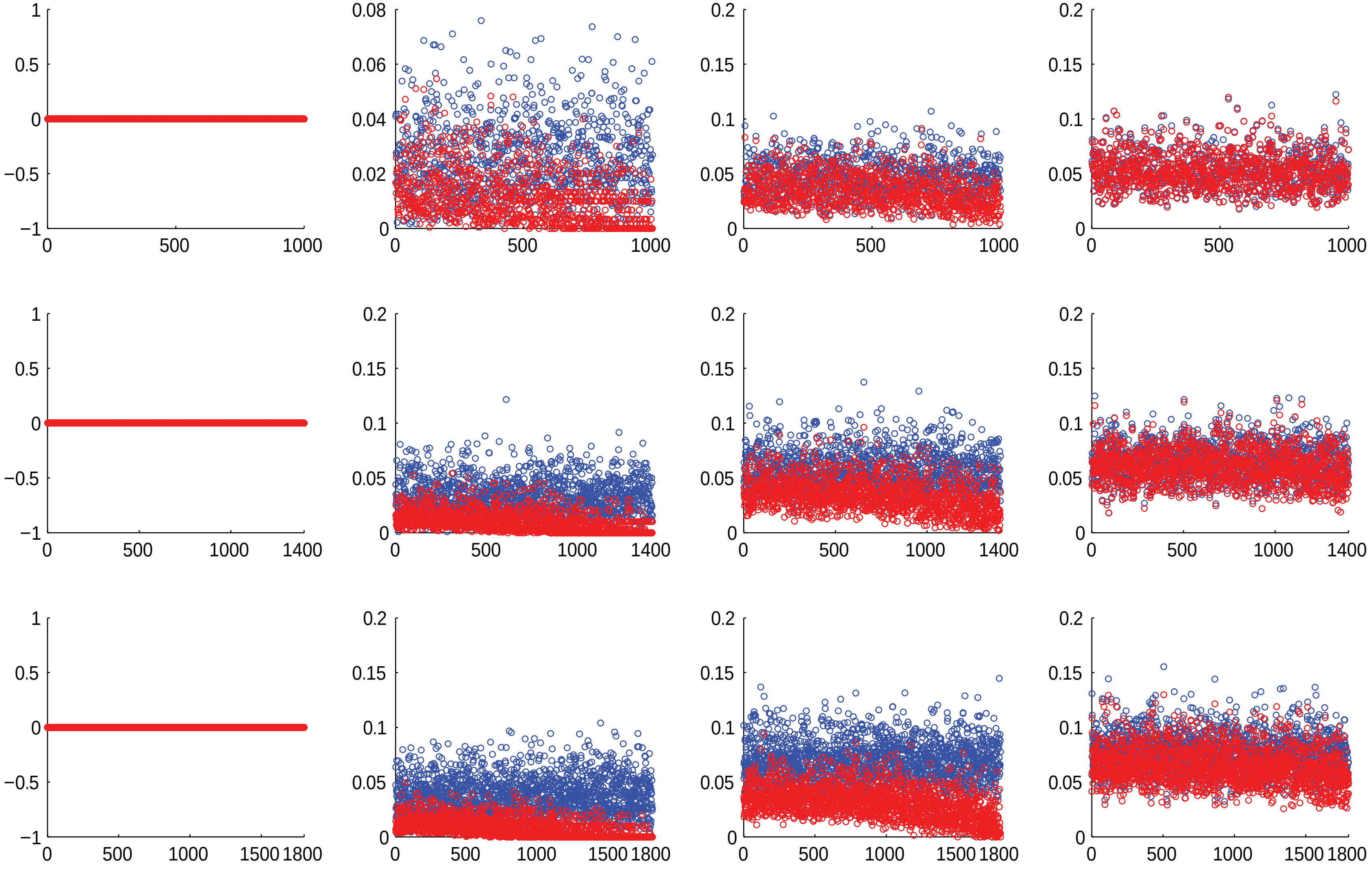

Figure A5. Local efficiency Red: serial growth, Blue: parallel growth,
x-axis: the sequence (order) of neurons starting to grow axons, y-axis: out-degree, from left to right, the size of neurons increases from 0.5 to 0.9 (0.500, 0.604, 0.735, 0.900) and also the maximum number of incoming connections increases. From top to bottom, the number of neurons increases from 1000 to 1800 by steps of 400. For serial growth, neurons started growing axons early on were characterized by slightly higher local efficiency than late starter neurons, whereas the order of starting did not influence local efficiency distribution for all neurons. The local efficiency for parallel growth was in general higher than the local efficiency for serial growth.The discrepancy of local efficiency between serial and parallel growth became less obvious for bigger neurons with more incoming connections. When neurons were allowed to accommodate only one incoming connections (1st column), all neurons had zero local efficiency, indicating all immediate neighbor neurons were disconnected.

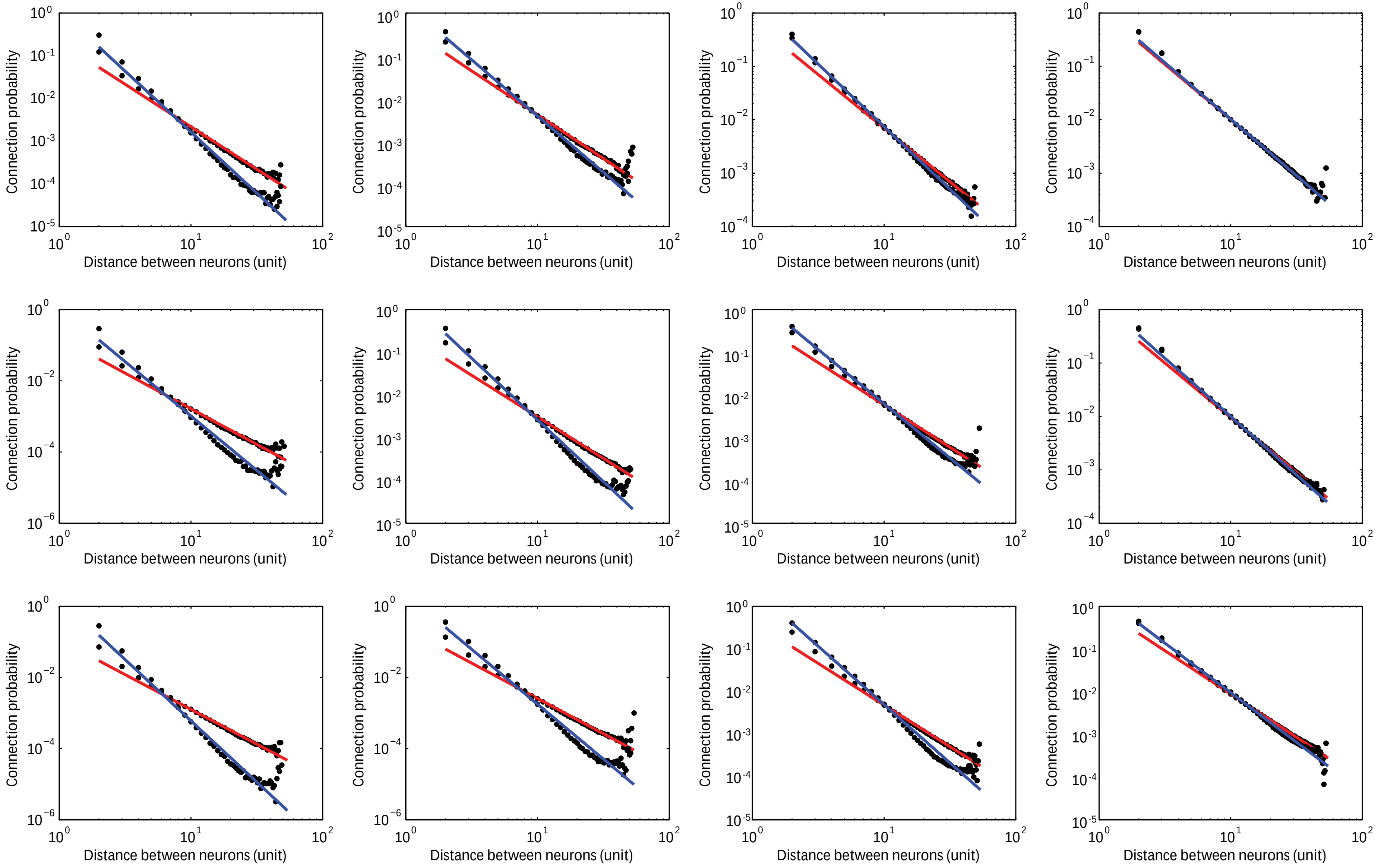

Figure A6. Connection probability: Serial vs. parallel growth. Doubly logarithmic plot (log-log plot), red: serial, blue: parallel, x-axis: distance between neurons (unit),y-axis: connection probability, From left to right: the size of neuron increased from 0,5 to 0,9 and from top to bottom: the number of neurons increases from 1000 to 1800 by steps of 400. Connection probability between two neurons at a given distance was inversely proportional to the distance between neurons. Connection probabiliy decreased more rapidly for parallel growth than for serial growth with distance; two neurons with a shorter distance are more likely to be connected for parallel growth. The discrepancy between serial and parallel growth became less obvious with the size of neuron and the number of incoming connections.

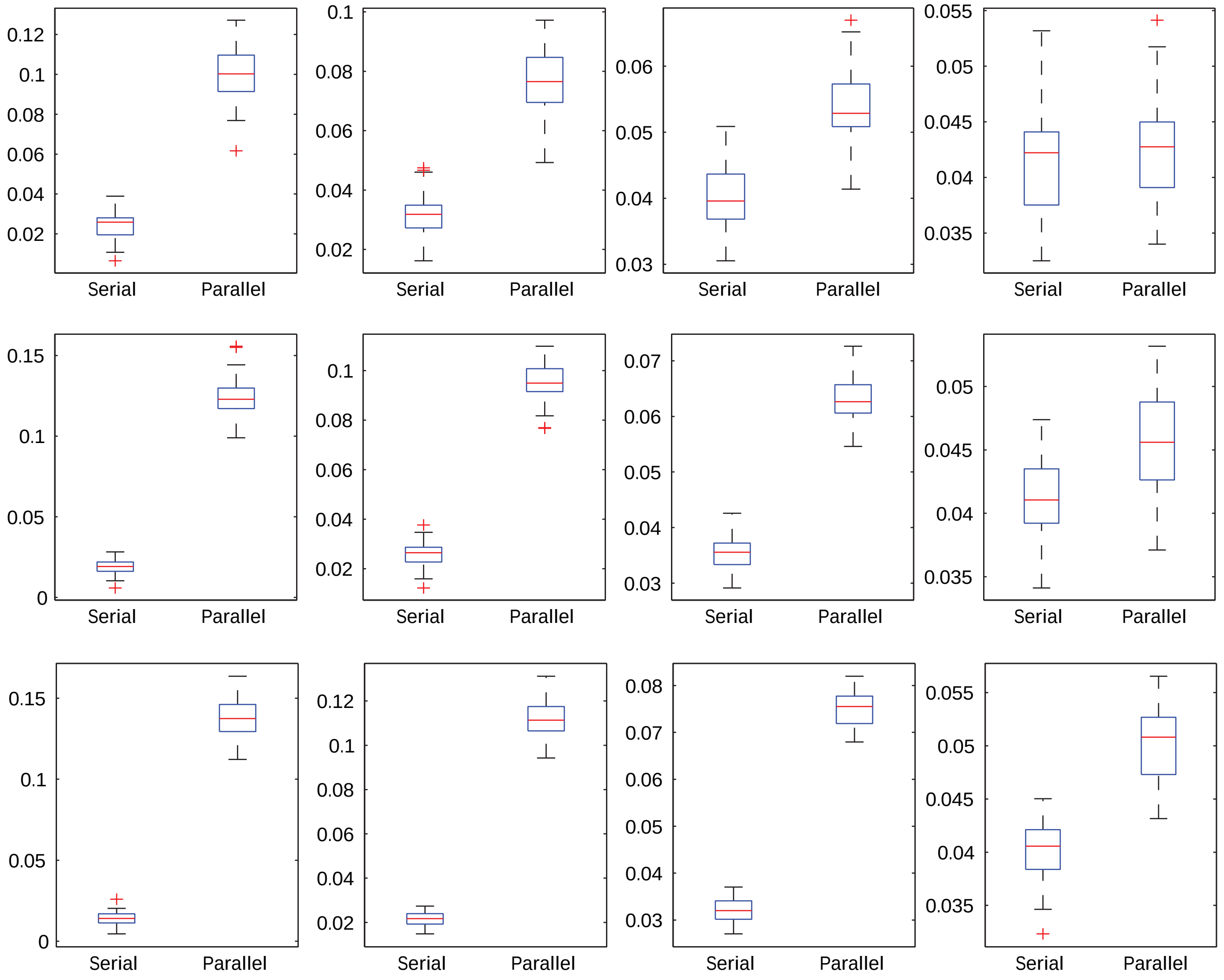

Figure A7 . Percentage of bidirectional connections with boxplot: Serial vs. parallel growth. x-axis: serial (left) and parallel (right), y-axis: the proportion of bidirectional connectivity, From left to right: the size of neuron increased from 0,5 to 0,9 and from top to bottom: the number of neuronsincreases from 1000 to 1800 by steps of 400. The bidirectional connections were more frequent for parallel growth than for serial growth. The discrepancy between serial and parallel growth became less obvious with the size of neuron and the number of incoming connections.

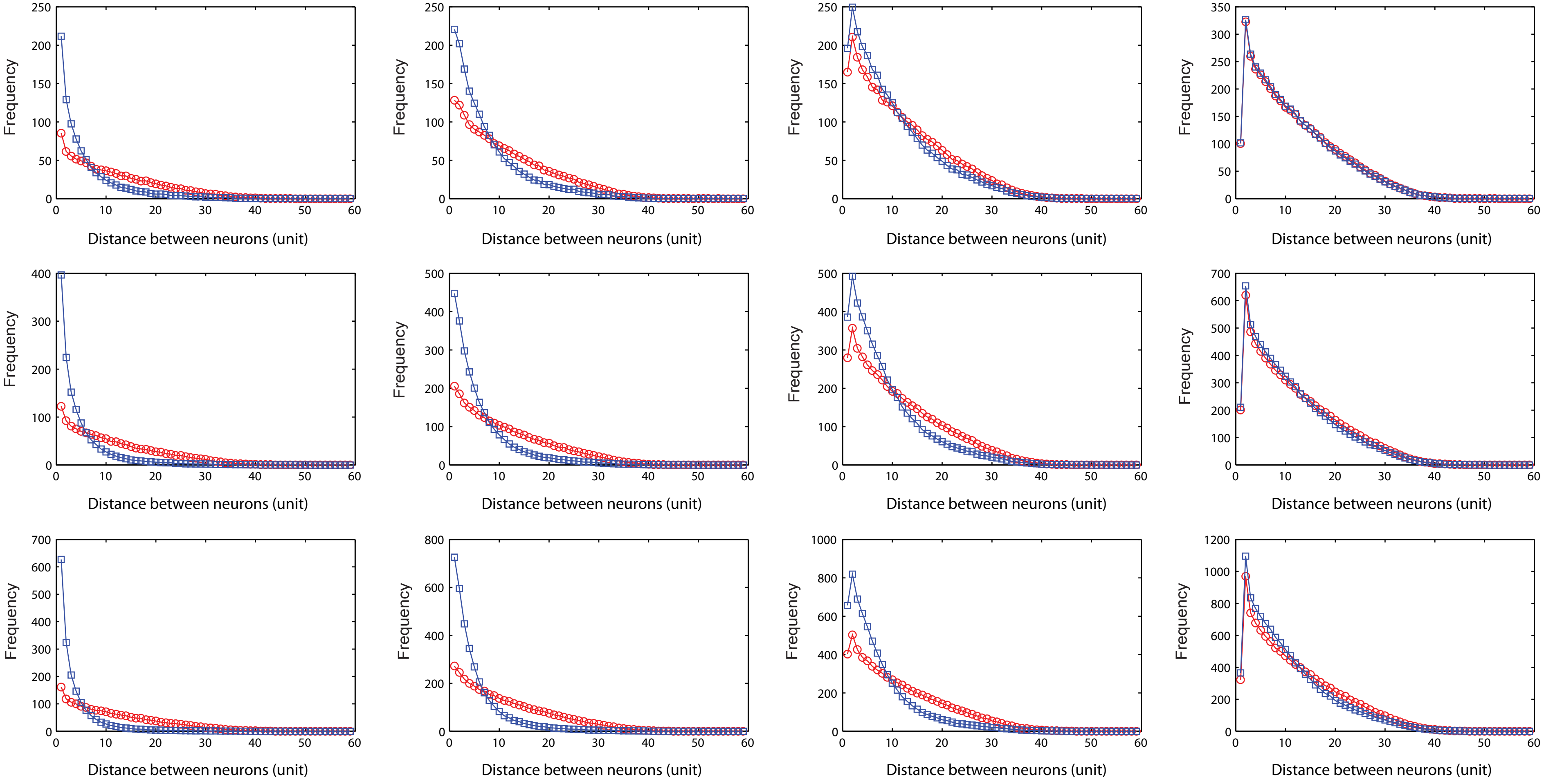

Figure A8. Connection length distribution. red: serial growth, blue: parallel growth x-axis: distance between neurons (unit), y-axis: connection probability, From left to right, the size of neuron increased from 0,5 to 0,9 From top to bottom, the number of neurons increases from 1000 to 1800 by steps of 400. For parallel growth, there were larger number of shorter connections whereas for serial growth there were less shorter connections and the proportion of longer connections was higher than for parallel growth. The discrepancy between serial and parallel growth in the connection length distribution became less obvious and almost no difference for larger size of neurons withi more incoming connection.

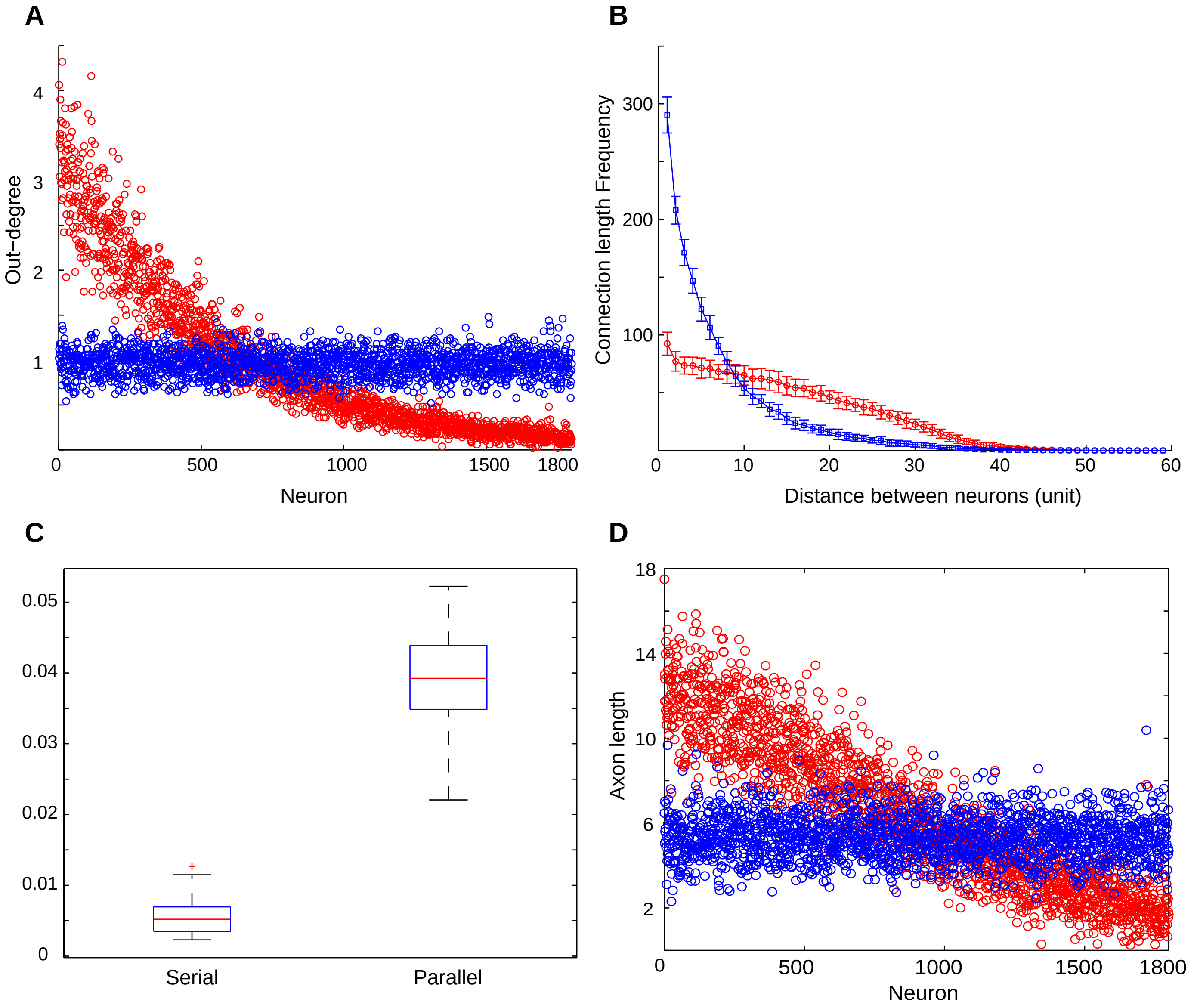

Figure A9. Simulation results with dendritic development. **A.** Out-degree, red: serial growth, blue: parallel growth, x-axis: for serial growth- the order of growth, for parallel growth- indices of neurons. **B.** Connection length distribution with distance between neurons. Red: serial growth, blue: parallel growth, **C.** Bidirectional connection ratio. Left: serial growth, right: parallel growth. The results were consistent with our previous results. **D.** Axon length. red: serial growth, blue: parallel growth, x-axis: for serial growth- the order of growth, for parallel growth- indices of neurons

A10. Partially overlapping time windows for axon outgrowth.

We additionally examined partially overlapping time windows for axon growth with groups of neuron growing together. We tested a small partial overlap and a large partial overlap; neurons were grouped into 2, 3 and 4 groups and were assigned to start growing axons after neurons in earlier starting group have elongated five (larger overlap) and ten (smaller overlap) unit length, in other words, neurons in other groups wait for 5 or 10 unit time. For instance, we divide neurons into two groups randomly and after neurons in the group start growing axons for 5 unit length, neurons in the other group start developing their axons. Serial growth is the extreme case of small overlap i.e. zero overlap and parallel growth is the opposite end where time windows of axon growth for neurons are maximally overlapped. Likewise, if we divide neurons into many groups, say into the same number of neurons, we would expected the smooth decrease in degrees and connection lengths, which was observed in main figures (Figure 2A and Figure 6B, respectively) where single neuron is actively growing its axon rather than multiple neurons in our main results. Earlier starting groups achieved higher out-degrees indicating better chances of becoming hub nodes in the networks; larger overlap of time windows produced more reciprocal connections between neurons. In summary, the results were consistent with the previous findings.

Figure A10. Degree distribution. 1st row: a large overlap in time windows for axon growth; 2nd row: a small overlap in time windows; column shows the number of groups in which neurons were divided. X-axis: neurons are orderly grouped according to their group time windows; Y-axis: out-degree. The discrepancy of out-degrees between groups is smaller with a large overlap than a small overlap case and earlier starting groups acquired higher out-degrees.

Figure A11. Axon lengths. 1st row: a large overlap in time windows for axon growth; 2nd row: a small overlap in time windows; column shows the number of groups in which neurons were divided. X-axis: neurons are orderly grouped according to their group time windows; Y-axis: Axon length. The discrepancy of axon length between groups is smaller with a large overlap than a small overlap case and earlier starting groups acquired longer axon lengths.

Figure A12. Reciprocal connections. X-axis: (left) a large overlap (right) a small overlap; Y-axis: the number of bidirectional connections. The bidirectional connections in neurons having large-overlapping time windows were more frequent than those of neurons with small-overlapping time windows; with more heterogeneous neurons i.e. larger number of groups, the number of bidirectional connection decreased (from left to right column).

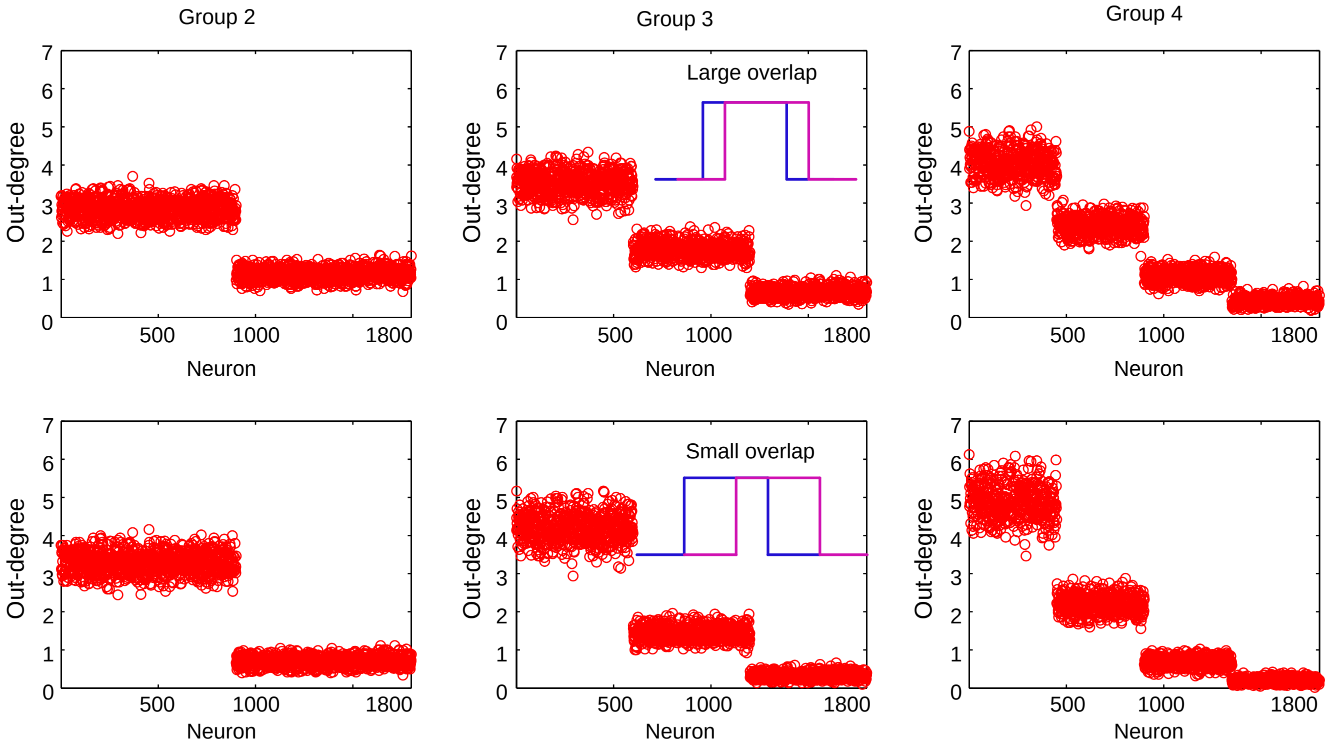

Group 2
Group 3
Group 4
Large overlap
Small overlap
Out-degree
Neuron
0
1
2
3
4
5
6
7
500
1000
1800

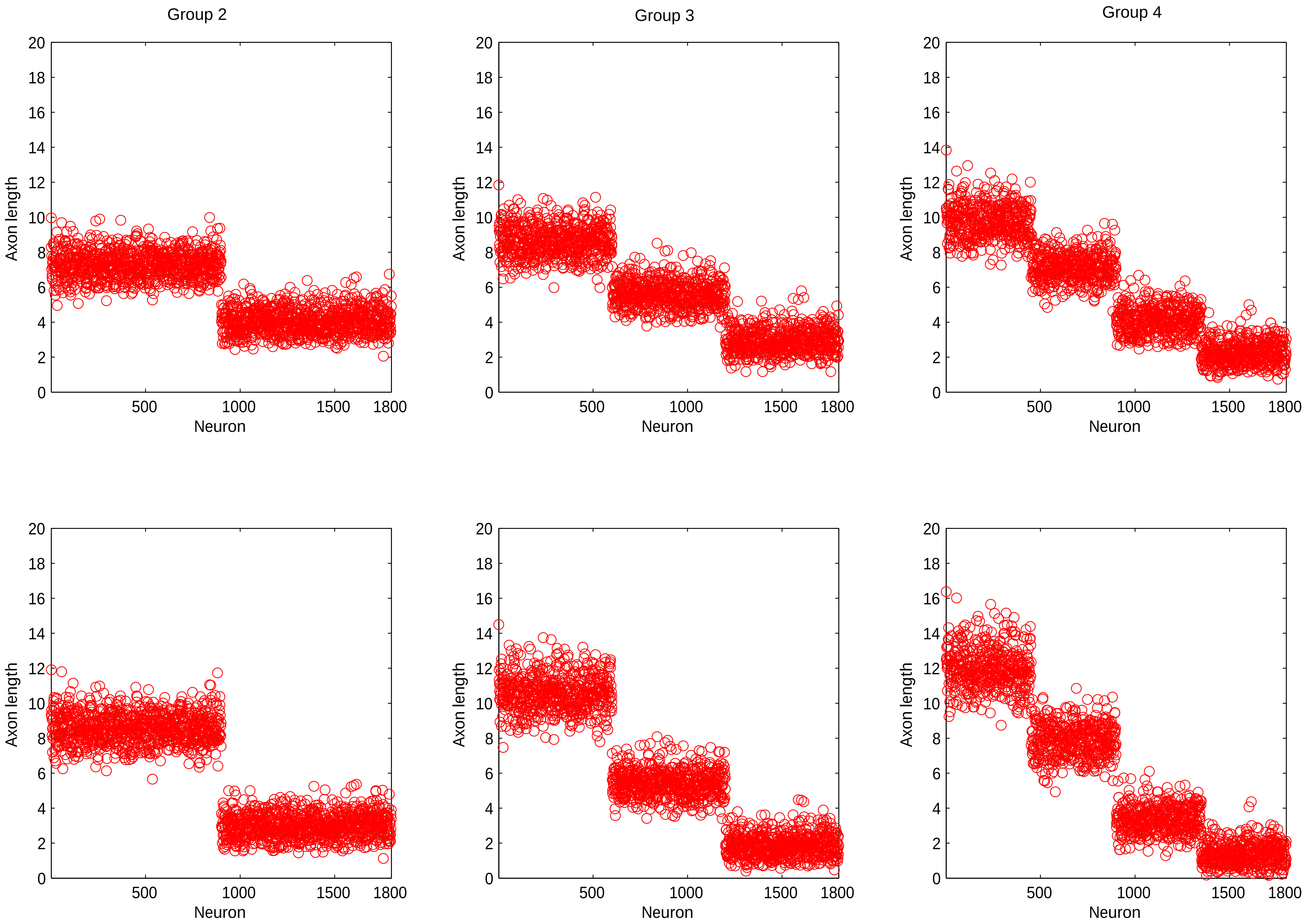
Group 2
Group 3
Group 4
Axon length
Neuron
0
2
4
6
8
10
12
14
16
18
20
500
1000
1500
1800

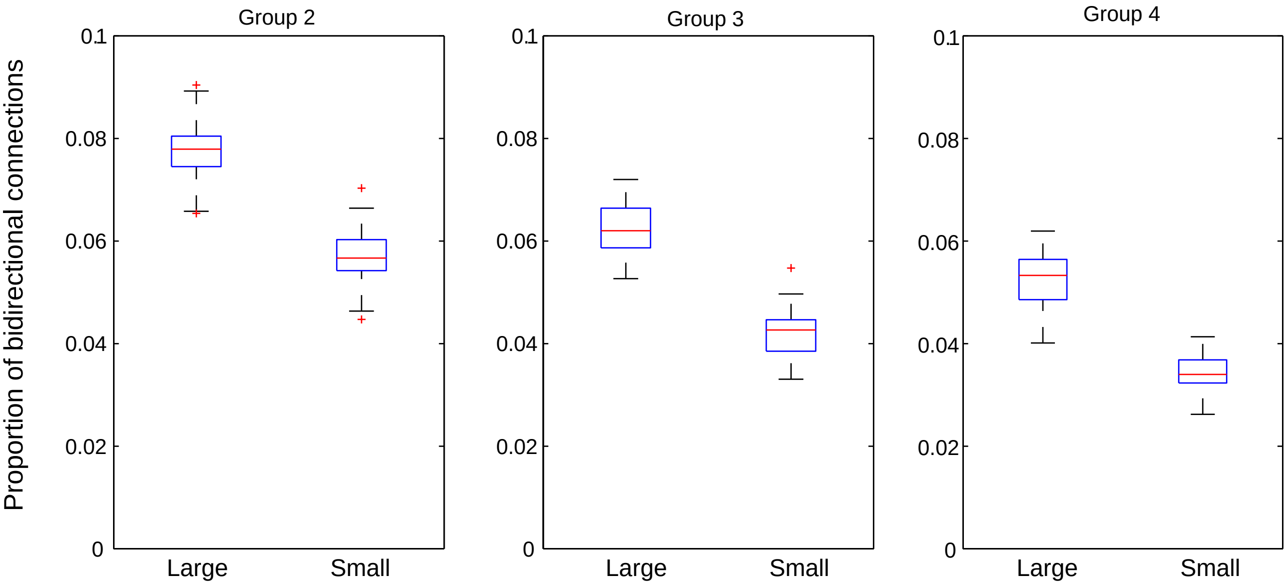

Group 2
Group 3
Group 4
Proportion of bidirectional connections
0.1
0.08
0.06
0.04
0.02
0
Large
Small

A13. Comparisons of local efficiency and connection probability from *C. elegans* data with the model predictions.

We calculated local efficiency and connection probability for *C.elegans* data. Unlike out-degree, bidirectional connections and axon length results in the main text, showed discrepancy from what the model predicted In particular, local efficiency results showed the opposite pattern from the model prediction; later-born neurons showed higher local efficiency than earlier-born neurons (Kruskal-Wallis test and post-hoc multiple comparisons were performed using Mann-Whitney test and corrected by Bonferroni , see methods in the main text) and connection probability as a function of distance did not show a simple exponential decrease (Figure A13). We believe that the model predictions and the actual results from *C.elegans* were different because i) differences in local efficiency between serial and parallel growth were less apparent and consistent as other measures in all conditions (Figure A5) and ii) connection probability in our model depends mainly on the geometrical arrangement of dendrites (neuron spheres) and axons, whereas the connectivity of *C.elegans* has additional constraints such as its elongated body shape and the higher prevalence for long-distance connections at the expense of having sub-optimal wiring cost to facilitate efficient information transfer in the network (Kaiser and Hilgetag 2006). Additional analysis of participation coefficient and within-module degree (Guimera and Amaral 2005) suggests that the higher local efficiency of late-born neurons were attributable to their lower within-module degrees and participation coefficients, which means late-born neurons were more connected within their modules resulting in higher local efficiency than earlier-born neurons (Figure A13c). Within-module degree and participation coefficient show nodal or local changes in modular organisation. Within-module degree indicates how well a node is connected to other nodes in the same module (Guimera and Amaral 2005); high within-module degree implies that the node is more connected to nodes within the module in which it participates than the average connectivity of the other nodes in the module. The participation coefficient indicates how well the node is connected to all other modules with higher values if many connections of the node are distributed to other modules.

Figure A13. A. Density distribution of local efficiency. Dashed line represents the median of the distribution, B. Boxplot of three birth time group and local efficiency, color scheme follows the main text. C. Participation coefficient and within-module degree Z, D. Connection probability as a function of distance.

A.

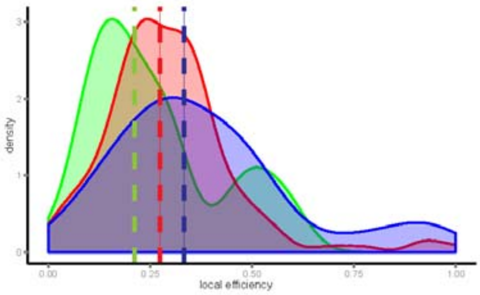

density
local efficiency

B.

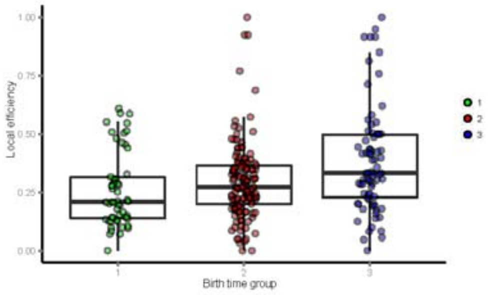

Local efficiency
Birth time group

C.

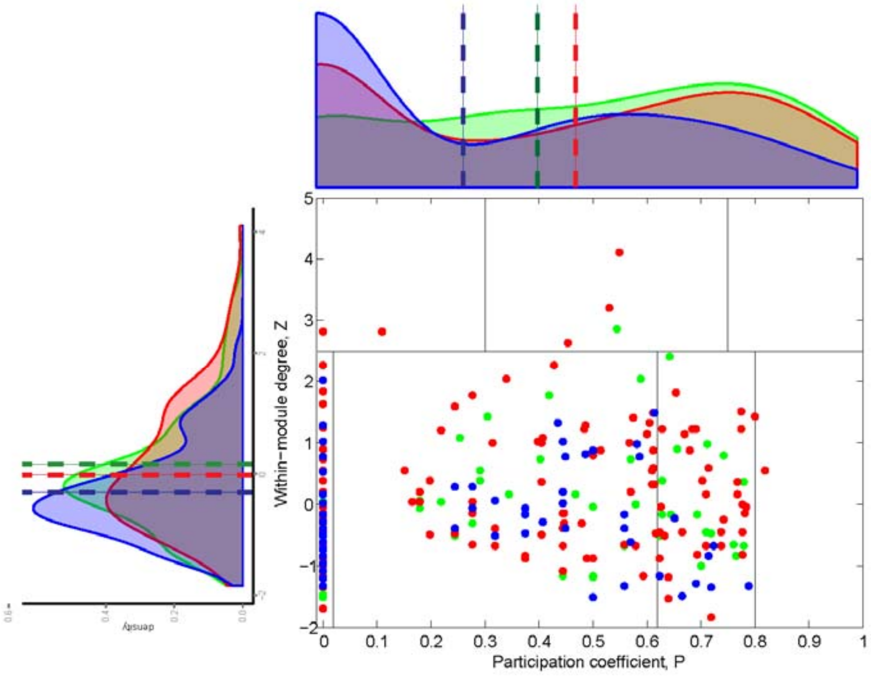

Within-module degree, Z
Participation coefficient, P
density

D.

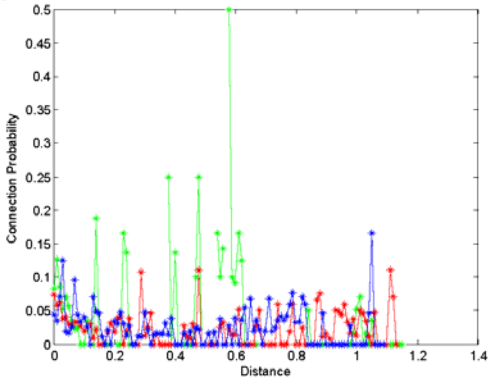

Connection Probability
Distance

A14. The effect on connection lengths when neurons change their position during development.

The embedding space of neurons for axon growth and synaptogenesis of our model was fixed during development, while internal volume changes through neurite growth and external mechanical factors could change the location of neurons and influence their synapse formation probabilities. To consider the effect on connection lengths when neurons change their positions during development, we need to consider the relative speed of axon and the movement (or migration) of neurons during development, what direction each neuron would choose to move or be forced to move during development due to the growth of the whole body for instance, whether neurons would still move their positions after they establish synapses with other neurons since it would affect the connection length between neurons, whether the growth direction of axons would change according to the position changes or not and so on. Thus, we start with the simplest condition for thought experiments and generalise to draw a conclusion.

For simplicity, let's assume that neurons can move their position in only one direction, e.g. x-axis of the reference frame and assume that the axon growth direction does not change. If the speed of axon growth is far faster than the movement of neurons, we do not need to worry about the effect on connection length because the changes of neurons' positions would be negligible. So the following scenarios assume that the speed of movement of neurons is not negligible compared to that of axon growth. Let neuron A grow its axons in the direction in the figure (Figure A14).

Let the position of neuron A at time t_1: $X(t_1) = (x_1, x_2, x_3)$, the position of neuron A at time t_2: $X(t_2) = (x_1+d_1t, x_2, x_3)$, the position of neuron B at time t_1: $Y(t_1) = (y_1, y_2, y_3)$ and the position of neuron B at time t_2: $Y(t_2) = (y_1+d_2t, y_2, y_3)$

, where t is the time passed ($t = t_2-t_1$) and d_1 and d_2 are the speeds in the x-axis direction of neuron A and B, respectively.

i) When $d_1 = d_2$, that is when all neurons have the same speed in the same direction (or the same velocity) to move their positions.

As both of the neurons moved along the x-axis with the same amount of displacement, the changes of positions of neurons do not affect the synaptogenesis; if neuron A were to establish a synapse with neuron B due to the close proximity between the growth cone and the neuron B, then A would make a synapse with neuron B if neuron B is available. The connection length between neuron A and B that are connected is defined by the Euclidean distance between the centres of neurons (Methods). The connection length is the same as before because the distance between the two neurons is the square

root of the sum of the position differences, which is equivalent with the connection length before they move to new positions. If those two neurons were not meant to be connected in the first place, which means that the axon growth cone would not find neuron B, then they would not be connected after both of them move their position in the same direction with the same amount of displacement for both neurons.

Connection length (distance between the two neurons' centres: $\|X(t_1) - Y(t_1)\| = \|X(t_2) - Y(t_2)\|$ when $d_1 = d_2$
θ and length l are the same, therefore $l\sin\theta$ is the same before they change their position, which means that if the growth cone of A is meant to find neuron B it will find it after both of them move laterally if B is available to accommodate another synapse.

ii) When $d_1 < d_2$ (assuming neuron A and B moves in the same direction),
If the growth cone could find neuron B in the vicinity (connectible range) in their original positions, after moving their positions the growth cone of neuron A may not find neuron B because both the distance between neuron A and B (l) and the sin θ increased, it is more likely for neuron B to be away from the connectible range.

iii) when $d_1 > d_2$, both l and $\sin\theta$ are decreased, if $l\sin\theta$ is less than the connectible range, neuron A can establish a synapse with neuron B when neuron B is available.

Now if neuron B does not move toward the same direction as neuron A, everything depends on the neuron B; regardless of the trajectory of neuron B's movement, when the position of neuron B at time t_2 : $Y(t_2)$ falls within the connectible range of growth cone of neuron A, neuron A can make a synapse with neuron B, otherwise there will be no synapse from neuron A to neuron B.

In summary, depending on the position of the connectible neuron's position relative to the position of the axon growth cone, either neurons can establish synapses or cannot make synapses. It will definitely change the results of the simulation; however, it will not change the qualitative differences between serial and parallel scenarios. If we assume that the neurons move their positions further apart from each other, the average connection lengths would be longer than that for the condition not assuming the expansion, nonetheless, the intrinsic nature of the characteristics of serial and parallel growth scenarios would remain the same.

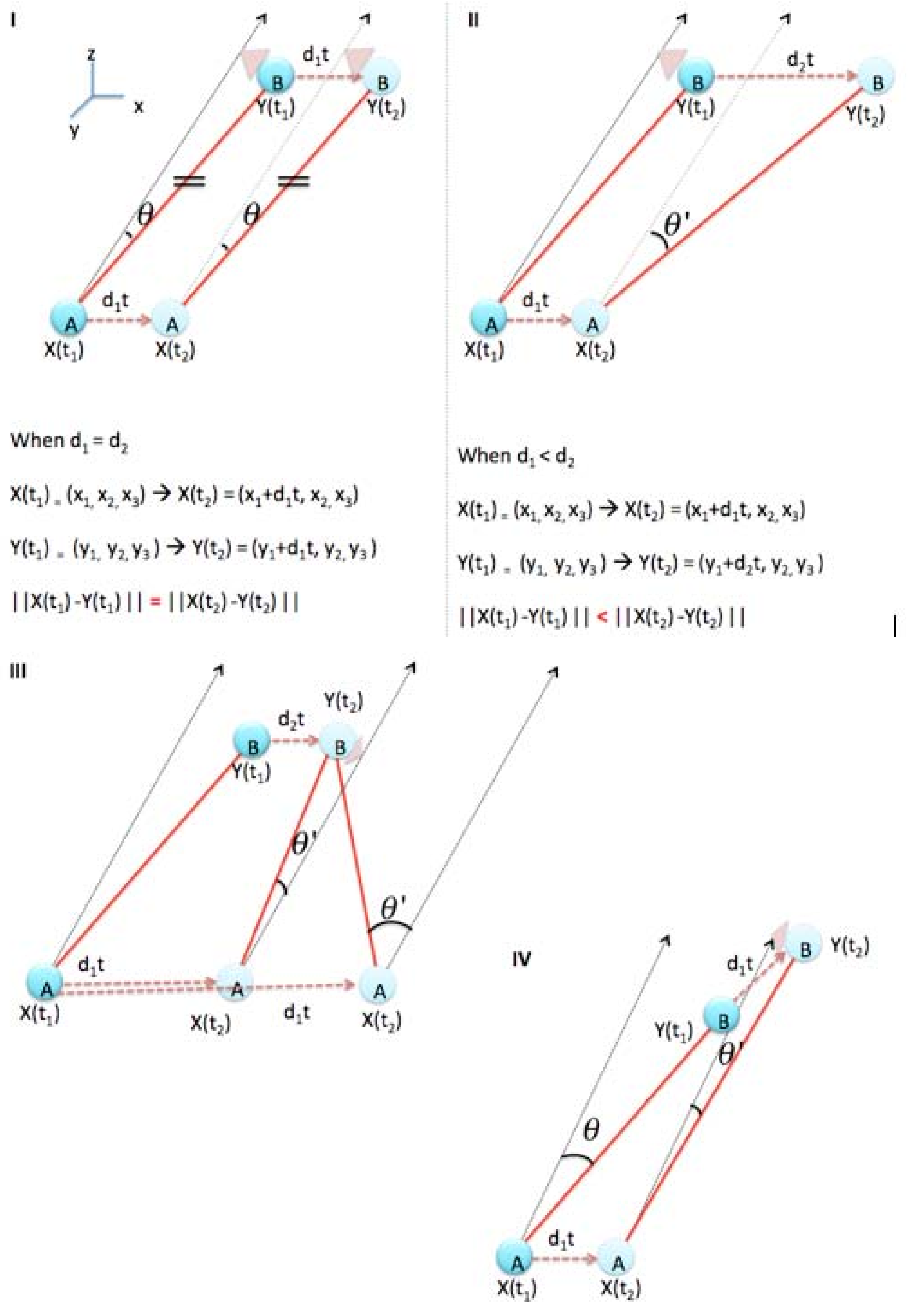

Figure A14. Representative cases to examine the effect on connection lengths when neurons change their position during development. Circles: neurons, solid red lines: connection lengths,

black dotted arrows: the growth direction for Neuron A, red dashed arrows: displacement in locations, triangles: synapses.
